# Evolution of platelet functions in cirrhotic patients undergoing liver transplantation: A prospective exploration over a month

**DOI:** 10.1371/journal.pone.0200364

**Published:** 2018-08-02

**Authors:** Daniel Eyraud, Ludovic Suner, Axelle Dupont, Christilla Bachelot-Loza, David M. Smadja, Dominique Helley, Sébastien Bertil, Ovidiu Gostian, Jean Szymezak, Yann Loncar, Louis Puybasset, Pascal Lebray, Corinne Vezinet, Jean-Christophe Vaillant, Benjamin Granger, Pascale Gaussem

**Affiliations:** 1 AP-HP, Pitié-Salpêtrière University Hospital, Department of Anesthesiology and Reanimation, Paris, France; 2 AP-HP, Pitié-Salpêtrière University Hospital, Department of Digestive, HPB Surgery, and Liver Transplantation, Paris, France; 3 Université Pierre et Marie Curie, Paris, France; 4 AP-HP, European University Hospital Georges Pompidou, Hematology Department, Paris, France; 5 AP-HP, Pitié-Salpêtrière University Hospital, Department of statistics, Clinical Research Unit, Paris, France; 6 Inserm UMR-S1140, Faculté de Pharmacie, Paris, France; 7 Université Paris Descartes, Sorbonne Paris Cité, Paris, France; 8 Inserm UMR-S970, Paris, France; 9 AP-HP, Pitié-Salpêtrière University Hospital, Hepatology Department, Paris, France; Institut d'Investigacions Biomediques de Barcelona, SPAIN

## Abstract

This prospective observational study was designed to analyze platelet functions across time in 50 patients scheduled for liver transplantation (LT) secondary to decompensated cirrhosis or hepatocellular carcinoma. Platelet functions were assessed before LT (pre-LT), one week (D7) and 1 month (D28) after LT. Platelet count significantly increased from pre-LT time to day 28 as well as circulating CD34+hematopoietic stem cells. To avoid any influence of platelet count on assays, platelet function was evaluated on platelet-rich-plasma adjusted to pre-LT platelet count. Although platelet secretion potential did not differ between time-points, as evaluated by the expression of CD62P upon strong activation, platelet aggregation in response to various agonists significantly increased along time, however with no concomitant increase of circulating markers of platelet activation: platelet microvesicles, platelet-leukocyte complexes, soluble CD40L and soluble CD62P. In the multivariate analysis, hepatic function was associated with platelet count and function. A lower platelet aggregation recovery was correlated with Child C score. History of thrombosis or bleeding was associated with respective higher or lower values of platelet aggregation. This longitudinal analysis of platelet functions in LT patients showed an improvement of platelet functions along time together with platelet count increase, with no evidence of platelet hyperactivation at any time-point.

## Introduction

Patients with end-stage liver disease, and especially those presenting with cirrhosis, have been considered for a long time at high risk of bleeding. Many factors sustained this opinion: the risk of perioperative bleeding is higher in end-stage liver disease [1], and liver disease is usually associated with multiple coagulation factor defects [2], thrombocytopenia [3,4], and thrombopathy [5,6]. Therefore, most of routine tests exploring coagulation, such as prothrombin time and activated partial thromboplastin time, are found abnormal in these patients. However, in recent papers, the reassessment of hemostasis in patients with chronic liver disease challenges the dogma that the major coagulopathy in these patients leads consistently to bleeding [7]. Other changes that accompany chronic liver disease may restore the balance of anticoagulant and pro coagulant effects and the main culprits for the bleeding tendency observed in patients with end stage liver disease should be sought among underlying conditions that favor hemorrhage, such as portal hypertension, endothelial dysfunction, bacterial infection, and renal failure in addition to the decrease in coagulation factors. Indeed, the defect of coagulation factor synthesis is compensated for by increased plasma levels of procoagulant factors such as FVIII, the inflammatory reactant von Willebrand factor (VWF) secreted by activated endothelial cells, or the simultaneous decrease of other factors of hepatic origin leading to a pro-coagulant state. It may concern ADAMTS 13, the ultra-large von Willebrand factor cleaving protease, the fibrinolytic precursor plasminogen, and/or the physiological coagulation inhibitors protein C and protein S. Moreover, while platelets were classically considered altered in count and in function [4,5], a recent study suggested that only cirrhotic patients with severe thrombocytopenia < 60x10^9^/L may present a limitation of thrombin generation [7]. Authors indeed showed that thrombin generation on platelet surface correlated with platelet numbers and was similar in patients and controls when samples were adjusted to the same platelet count. These findings support the hypothesis that thrombocytopenia, and not thrombopathy, plays a key role in thrombin generation and possibly bleeding tendency in patients who have cirrhosis. However, the multiple causes of thrombocytopenia and/or thrombopathy during chronic liver disease and the fact that there is not universally accepted platelet function assay in this clinical setting make challenging any firm conclusion. Anomalies of laboratory tests do not always imply a defect of platelet functions *in vivo*. For instance, other mechanisms might counterbalance platelet low count and/or dysfunction, such as an increase or platelet adhesion ability secondary to the presence of circulating VWF multimers of high molecular weight or to a decrease of hepatic clearance of FVIII and VWF[8,9]. On the other hand, platelet activation might result from other components present during cirrhosis, such as a high circulating level of LPS[10]. Other studies conversely found a defect in platelet functions before transplantation, in terms of reduced aggregation potential and reduced GPIIbIIIa (fibrinogen receptor) and P-selectin (CD62P, marker of alpha granule secretion), that was restored on the third day after reperfusion [9]. Thrombocytopenia has both central and peripheral origins, including a decrease in thrombopoietin synthesis by the liver, platelet sequestration in spleen, and consumption. Markers of platelet activation have been found increased in those patients, but these high levels are likely to result from a delayed clearance due to hepatic dysfunction and their significance is still a matter of debate as well as the clinical importance of markers of disseminated intravascular coagulation.

The present study was designed to monitor the evolution of platelet functions across time in end-stage liver cirrhosis patients before, one week and one month after liver transplantation. Our aim was to determine if an improvement of platelet functions in a given patient occurred concomitantly to platelet post-transplantation increase.

## Materials and methods

### Study population

The PLATON study was a prospective observational study, conducted from February 2010 to January 2013, which was approved by our institutional review board (Comité de Protection des Personnes, Hôpital Pitié-Salpêtrière—Paris VI—Ile de France). Because care of patients conformed to standard procedures currently used in our institute and because platelet and endothelial functions were measured in residual blood samples, waived informed consent was authorized. However, oral and written information was given to the patients.

All included patients had to be aged over 18 years and to be listed on the National register of liver transplantation for liver cirrhosis. Exclusion criteria were: age below 18, re-transplantation, multiple organ transplantations, fulminant hepatitis and patients for whom platelet function could not be assessed the day of inclusion. Cirrhosis was diagnosed based on clinical, laboratory, ultrasonographic and tomodensitometric or histologic evidence. Hepatocellular carcinoma (HC) was diagnosed on the basis of tomodensitometry, magnetic resonance imagery (MRI), ultrasonography and in some case histologic evidence. The severity of cirrhosis was estimated according to Child-Turcotte-Pugh classification [11], UNOS score and MELD score.

Liver transplantation (LT) was performed using total hepatectomy with preservation of the inferior vena cava, partial clamping of the native vena cava as described elsewhere [12]. Quality of liver graft was a miscellaneous criteria, assessed before LT, in relation with accepted “donor extended criteria”[12]. We differentiated liver graft quality into two classes: good or mediocre.

All the patients received a standard immunosuppressive regimen, which included prednisone, mycophenolate, and cyclosporine or tacrolimus.

### Blood samples procedure

Comprehensive biological testing was performed at three-time points in patients: at inclusion (pre-LT), then 7 and 28 days after reperfusion. Peripheral blood samples were collected into EDTA, 0.105 M citrate and lithium heparinate tubes. Routine coagulation parameters were determined on a STA-R analyzer (Stago, Asnières, France). References of reagents used are available upon request. Thrombin-antithrombin complexes were determined by ELISA (Abcam #108907, Cambridge, UK). Normal human plasma levels given by the manufacturer are 1.0–10 ng/mL.

### Liver function

Liver function was assessed by aspartate aminotransferase (ASAT) and alanine aminotransferase (ALAT), bilirubin (total and conjugated), albumin, and prothrombine time (PT), factor V before LT, daily after LT until day 5, at day 7, then according to evolution of the results and at day 28 after LT.

### History of thrombosis or bleeding

History of thrombosis was defined as one or more episodes of thrombosis, including portal thrombosis, lower limb deep venous thrombosis or pulmonary embolism. History of bleeding was defined as one or more episodes of bleeding, including esophageal varicose vein rupture, gastric bleeding.

### Functional platelet assessment

#### Platelet aggregation

Aggregation studies were performed within 3 hours after blood collection, on a TA-8V optical platelet aggregometer (Soderel Medical, Heillecourt, France). Platelet-rich plasma (PRP) was obtained by centrifugation at 200 g for 10 min, and the platelet count was not adjusted, as recommended [13]. Since our aim was to assess platelet function in a single patient over time, platelet counts in PRP of D7 and D28 samples were adjusted to that of pre-LT sample with autologous plasma. Aggregation tests were performed using the following agonists: 1.5 mM arachidonic acid (Helena Biosciences Europe, Saint-Leu La Forêt, France), 1 μg/ml Horm collagen (Nycomed Pharma, Paris, France), 5 and 10 μM ADP (Biopool, Umea, Sweden), 1 mg/mL ristocetin (Helena Biosciences) and 20 μM thrombin receptor 1 activating peptide (TRAP6, Stago, Asnières, France). Platelet aggregation was recorded for 8 minutes and expressed as maximal aggregation (%).

#### Platelet secretion

The potential of platelets to secrete their granule contents was evaluated *in vitro* by flow cytometry by the expression level of P-selectin (CD62-P) on platelet surface, a specific marker of alpha granule, upon TRAP-induced activation [14]. Briefly, all experiments were performed in the presence of Integrilin (10 μg.mL^-1^ final, GlaxoSmithKline, Marly-le-Roi, France) to avoid platelet aggregation. PRP was incubated with buffered saline (PBS) or TRAP6 (50 μM) for 10 min at room temperature. Measurement of P-selectin (CD62P) expression before and after platelet activation was performed after incubation in the dark for 10 min with anti-CD62P-PE (Beckman Coulter, Villepinte, France) or mouse immunoglobulin IgG1-PE. After incubation, a solution of PBS-PFA 0.5% was added to stop the reaction. Results were expressed as the % of activated platelets in the presence of TRAP. Activated platelets at rest (PBS) were below 10%.

#### Platelet-leukocyte complexes

Circulating activated platelet level was evaluated *ex vivo* by activated platelet ability to form so-called platelet-leukocyte complexes (PLC). Whole-blood PLC were counted by flow cytometry [15]. The samples were analyzed with a FACScalibur device (Becton-Dickinson, Le Pont de Claix, France). PLC were identified as the cell population stained with both anti-CD41-PE (Beckman Coulter) and anti-CD45-FITC (Beckman Coulter) and expressed as a percentage of total leukocytes.

#### Platelet microvesicles

Platelet-poor plasma (PPP) was obtained from blood samples on citrate after one centrifugation at 1500 g during 15 minutes at 20°C, followed by 13000g during 2 minutes at 20°C. PPP were frozen at -80°C until use. Platelet microvesicles (PMV) were quantified by flow cytometry, using CD41-PE monoclonal antibody (Beckman Coulter) and Annexin V-FITC (Becton Dickinson) in the presence or in the absence of annexin V binding buffer (Becton Dickinson). Flow count beads (Beckman Coulter) were added to each sample to calculate the absolute number of microvesicles. Samples were analyzed on a Navios flow cytometer using Kaluza software (Beckman Coulter). PMVs present in plasma were analyzed according to their size and fluorescence. Each sample was analyzed in duplicate. Results were expressed as the total number of PMVs annexin V+ per microliter of plasma and as a percentage of total platelet count.

#### Other markers of platelet activation

Soluble CD40L was determined using the ELISA kit from Invitrogen (Thermo Fischer Scientific, Waltham, MA, USA) according to the manufacturer’s instructions. Normal human plasma levels given by the manufacturer are 0.03–3.98 ng/mL.

### Statistical analysis

Correlation between longitudinal data was explored 2 by 2. To assess the between subject component of the correlation, measuring the correlation between (true) subject means, we measured a weighted correlation coefficient, using the number of observations, repeated measures, as weights, as there are various numbers of observations per subject. It indicates whether subjects with high values of one variable also tend to have high values of the other. The within-subject correlation describes whether an increase in one variable within an individual is associated with an increase in the other. The second component of the correlation between the two variables was done using analysis of covariance, by eliminating the subject effect [16,17].

To explore the time evolution and factor associated with the evolution, a linear mixed model with subject random intercepts was used to assess the time effect (linear and quadratic) on repeated measures of parameters. To select factor associated with the evolution of parameters, analysis model resulted from a classical 2-step analysis plan. We tested the associations between different covariates and repeated measures of various parameters (1 × 1). In the multivariate model, only factors that attained statistical significance (*p-value* < 0.15) in the first step were included. According the number of tests (correlation between longitudinal data, time evolution and factors associated with the evolution) a *p-value* < 0.0001 was considered statistically significant (Bonferroni correction). The data analysis was performed with R version 3. 1.3 for Windows.

Aggregations results at D28 were analyzed in a subgroup of 28 among the 50 patients having platelet count in PRP above 150 G/L, by comparison to the in-lab reference values obtained in 48 healthy controls. To assess the differences between maximal aggregation values, Wilcoxon rank sum test was used and p-values were adjusted for multiple comparisons (Bonferroni method).

## Results

### Patients

Consecutive patients fulfilling inclusion criteria were enrolled during a 36-month period among 101 patients scheduled for LT fulfilling the inclusion criteria.

**Fig 1:**
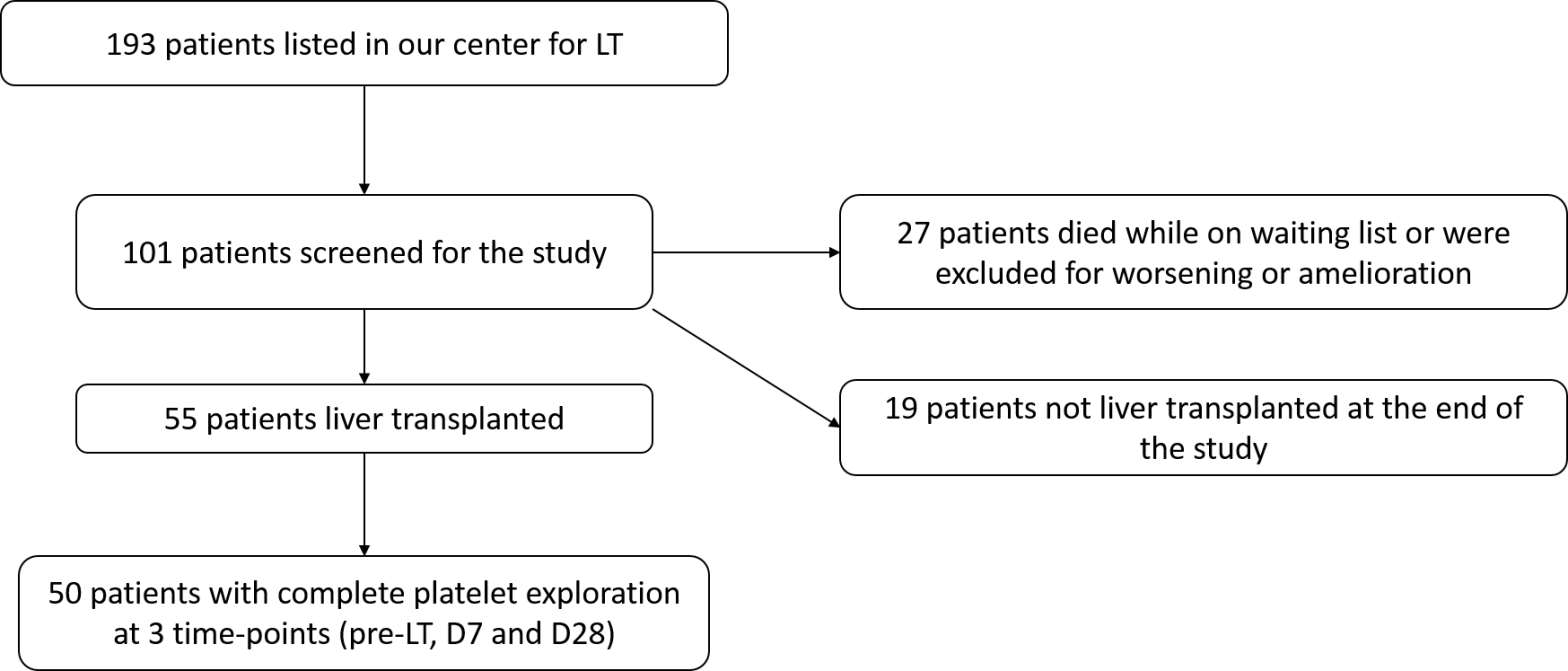
Flow chart. LT: Liver transplantation.

As summarized in Fig 1, among 55 patients included, 50 patients with complete platelet exploration at the 3 time-points (25.9% of those initially screened) (42 men, 8 women) with a median age of 59 [53;62] years were enrolled. Patients’ demographic characteristics, severity of cirrhosis, history of bleeding or thrombosis and quality of the liver graft are given in Table 1.

**Table 1:** Baseline characteristics of the study population at inclusion and quality of the liver graft.

Characteristics	n = 50
Sex(Male/Female	42/8
Age (yr)	59 [53;62]
Indication of liver transplantation (HC/ viral or alcoholic cirrhosis/ other)(n)	24/23/3
UnosUNOS classification (1/2/3/4) (n)	15/3/3/29
Severity of the cirrhosis (Child-Turcotte-Pugh A/B/C) (n)	15/9/26
MELD score	20 [14;25]
History of thrombosis (yes) (n)	9
History of bleeding (yes) (n)	20
Quality of the liver graft (good) (n)	39

Quantitative data are given as median values [Q1;Q3], qualitative data are expressed as frequency.

During the duration of the study, in the cohort of transplanted patients, 3 had deep venous thrombosis at respective days of 35, 44 and 51 post-transplantation, and were treated with heparin. One patient experienced a bleeding event two days after transplantation (platelet count 75 x 10^9^/L) and was operated again. The median intraoperative blood transfusion was 6 [4;10] red blood cell packs. The time of cold ischemia was 505 [445;604] min and the time of warm ischemia was 52 [39;89] min.

All included patients were alive at day 30 but 3 died from infection during their hospitalization at day 67, and at months 2 and 6 after the LT.

As expected, a significant decrease in bilirubin and ALAT levels was found after reperfusion together with concomitant improvement in prothrombin time, factor V and fibrinogen level (p < .0001 for all parameters, Table 2). Thrombin-antithrombin complexes (TAT) quantified in a subgroup of 27 patients showed a significant evolution across time with a linear and quadratic effect. A significant decrease was observed along time with a plateau reached at D28 (Table 2).

**Table 2:** Baseline and evolution of the biological parameters of the 50 patients with the 3 time-point available data.

Parameters		pre-LT	D 7	D 28
Platelets (10^9^/L)*		70.5 [51;119]	88 [54.3;122.5]	204 [136;298]
Leukocytes (10^9^/L)		4.7 [3.5;5.9]	7.5 [5;10.5]	6.1 [4.7;7.2]
Hemoglobin (g/dL)		10.1 [8.8;12.1]	11.1 [9.8;12.5]	10.9 [9.8;11.9]
Total bilirubin (μM)*		54 [29;128]	35 [24;65]	15 [9;21]
Conjugated bilirubin (μM)*		30 [16;68]	22 [14.5;45]	16.5 [12.8;21]
Creatinine (μM)		69.5 [59.3;97.5	65 [46.8;85]	77.5 [63.5;94.5]
Alanine aminotransferase (UI/L)*		183 [41;327.8]	89 [53.5;165.8]	23.5 [14.8;34.8]
Prothrombin time*$		30 [22.8;46]	81 [71;90.8]	90 [80;96]
Factor V (%)*$		26.5 [18;37]	61.5 [50.5;90]	93 [82.5;103]
Fibrinogen (g/L)*$		1.6 [1.2;2.4]	3.4 [2.92;4.2]	4.45 [3.8;5.3]
TAT complexes (ng/mL)*$		39.79 [30.7;62]	32.22 [17.21;44.04]	28.82 [16.08;50.79]
Platelet aggregation (%)	5μM ADP*	42 [16;62.5]	49 [23;71]	65.5 [52;75.8]
	10μM ADP*	58 [32.5;71.5]	61 [45;75]	73 [62.5;78.8]
	20μM TRAP	48 [19.5;71]	50 [28;73]	61 [32.5;73]
	1.5 mM Arac acid	19 [8;58.5]	48 [17;65.3]	68 [41;75]
	1 μg/mL Collagen	39 [14;59]	31 [17.5;68.5]	59.5 [25.3;72.8]
Platelet agglutination (%)	1 mg/mL Ristocetin	63 [29.5;84.5]	74 [56;91]	78 [60.3;88]
Platelet Microvesicles annexinV positive	/μL plasma	199 [79 ;357]	185 [97 ;348]	514 [193;980.3]
	%/total platelet count	0.26 [0.11 ;0.56]	0.2 [0.12 ;0.47]	0.22 [0.1 ;0.46]
CD62P positive platelets (%)	TRAP activation	89.7 [79.3;94.7]	84.1 [76.8;89.4]	89.5 [82.9;93.2]
Platelet-Leukocyte complexes (%)		20.7 [11.9;29.3]	13.7 [7.7;21]	25.7 [14.5;35]
CD40L (ng/mL) *$		0.89 [0.48;1.39]	0.46 [0.24;0.62]	0.88 [0.5;1.27]
sCD62P (ng/mL)		2.1 [1. 6;2.9]	1.4 [1.2;2]	1.7 [1.4;2.2]
CD34+ cells (/mL)		765 [385;1255]	810 [330;1655]	1575 [987.5;2875]

Data are expressed as median values [1^st^-3^rd^ interquartiles]

*significant linear time effect (p-value < 0.0001)

$ significant quadratic time effect (p-value < 0.0001).

TAT and CD40L were performed in a subgroup of 27 patients.

### Platelet count and functions

#### Platelet count

**Fig 2:**
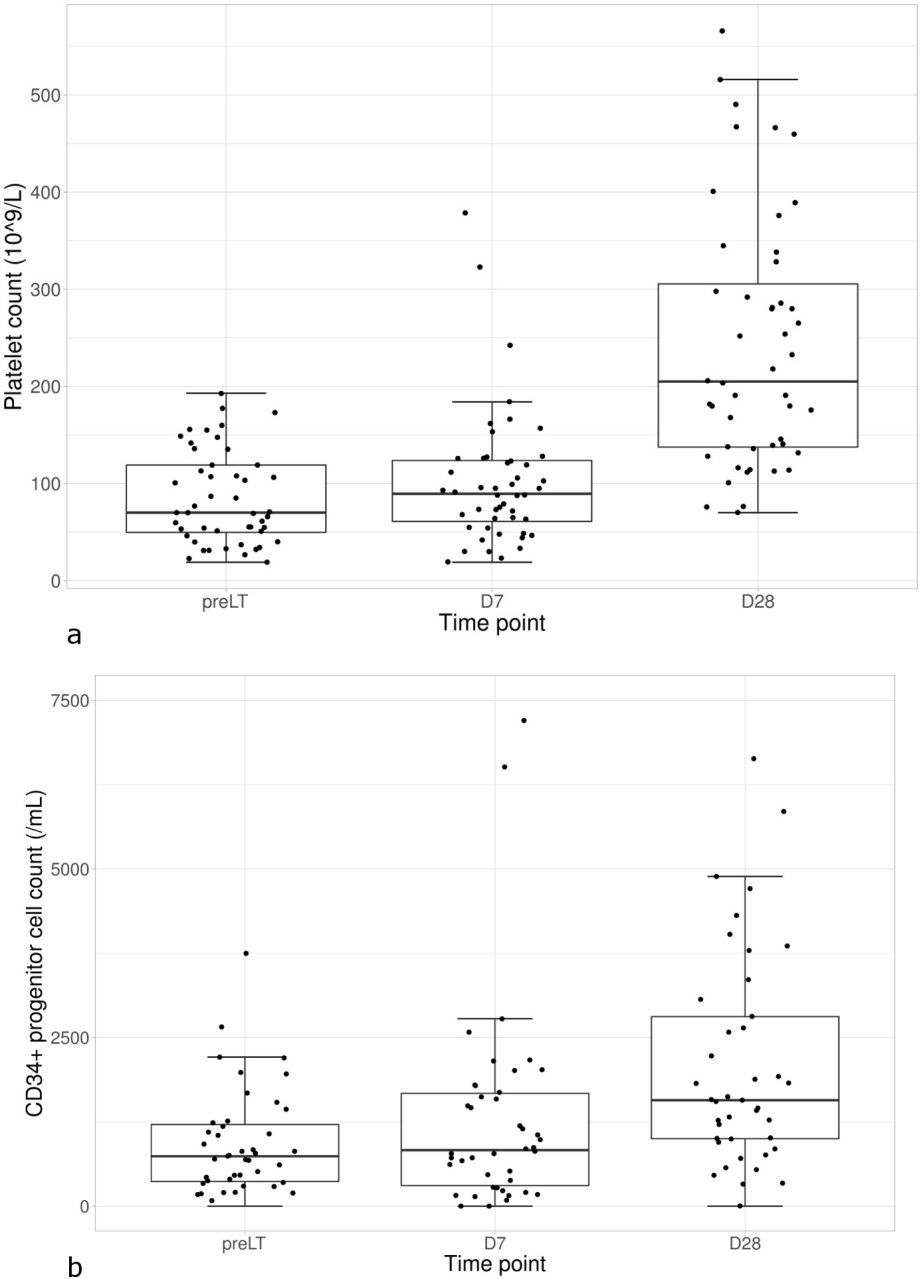
**(A) Boxplots for platelet count and (B) CD34+ hematopoietic progenitor stem cells at different time points.** Horizontal lines show median values and the 25–75 percentiles. Pre LT: pre liver transplantation.

Platelet count significantly increased from pre-LT time to D28 to reach a normal median count of 204.10^9^/L (Table 2). Only 3 patients remained below 100.10^9^/L 28 days after transplantation (Fig 2A). None had values below 70.10^9^/L at D28. Along with recovery of liver function and bone marrow platelet regeneration, circulating CD34+hematopoietic stem cells gradually increased in number (Table 2 and Fig 2B).

#### Platelet aggregation in response to various agonists

**Fig 3:**
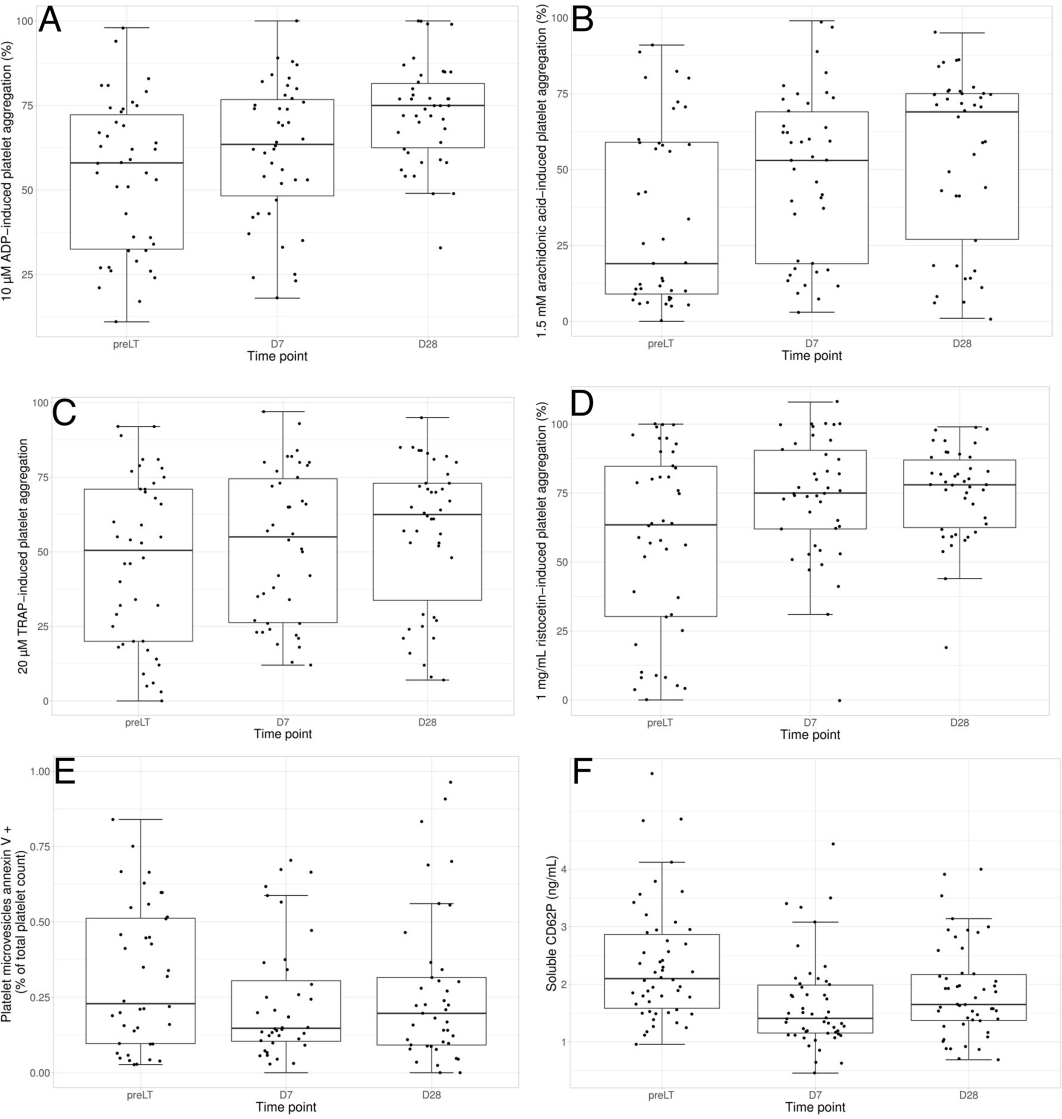
**Boxplots at different time points for (A) 10 μM ADP-induced platelet aggregation, (B) 1.5 mM arachidonic acid-induced platelet aggregation, (C) 20 μM TRAP-induced platelet aggregation, (D) 1 mg/mL ristocetin-induced platelet agglutination, (E) platelet microvesicles as a % of total platelet count, (F) soluble CD62P.** Horizontal lines show median values and the 25–75 percentiles. Pre LT: pre liver transplantation.

Noteworthy, our aim was to assess platelet function in a single patient over time. Therefore, platelet counts in PRP of D7 and D28 samples were adjusted to that of pre-LT sample with autologous plasma. Platelet aggregation in response to various agonists increased with time. For instance, median values for maximal aggregation induced by 10 μM ADP were 58%, 61% and 73% for pre-LT, D7 and D28, respectively (Fig 3A and Table 2, p<0.0001). Same feature was found in a lesser extent for other agonists, such as arachidonic acid, TRAP and agglutination in response to ristocetin (Fig 3B–3D and Table 2).

Overall, an improvement of platelet aggregation was found. However, when comparing platelet aggregations of 48 healthy controls to the subgroup of patients having a normal platelet count at D28 (N = 28 among the 50 patients with platelet count > 150 G/L in platelet-rich plasma), we found a significant difference in maximal platelet aggregation (S1 Table), as well as in residual aggregation (data not shown), whatever the agonist used. No significant difference in platelet counts was denoted at D28 between the populations with aggregations above or below 50%. In contrast, we found a significant association at pre-LT between platelet count and platelet aggregation.

#### Other platelet function tests

Platelet aggregation does not always reflect function, especially in the current clinical setting. Indeed, results in PRP might be influenced by platelet count or plasma content, especially in the samples obtained before transplantation. We therefore used other recognized tests to evaluate platelet functionality. By flow cytometry, we evaluated the potential of platelets to secrete their granule contents upon activation. The percentage of platelets expressing surface CD62-P, a specific marker of alpha granule membrane, quantified in response to the peptide agonist of the thrombin receptor (TRAP) did not differ according to the 3-time points, suggesting a normal secretion upon strong activation.

We further quantified circulating platelet microvesicles (PMV). Microvesicles are sub-micron-sized vesicles released by blebbing from membranes in response to activation or apoptosis. The large majority of circulating MV originates from platelets (PMV). Their surface is procoagulant subsequent to the externalization of phosphatidylserine to the outer leaflet of the platelet membrane, evidenced by the binding of annexin V. PMV when expressed as a percentage of the total platelet count did not increase along time (Table 2 and Fig 3E).

There was no evidence for an increase of *in vivo* circulating activated platelets as illustrated by the non-significant increased level of platelet-leukocyte complexes (PLC), despite the huge increase in platelet count after transplantation (Table 2). Moreover, soluble CD62P, a recognized circulating marker of *in vivo* platelet activation, tended to decrease between pre-LT and D28, despite the 3-fold increase in platelet count (Table 2 and Fig 3F). Finally, the soluble CD40L level, a specific marker of platelet activation, was quantified in a subgroup of 27 patients. A significant evolution across time was observed with a decrease at D7 and a further increase at D28 to reach the same levels as pre-LT values (Table 2, linear and quadratic effect).

**Fig 4:**
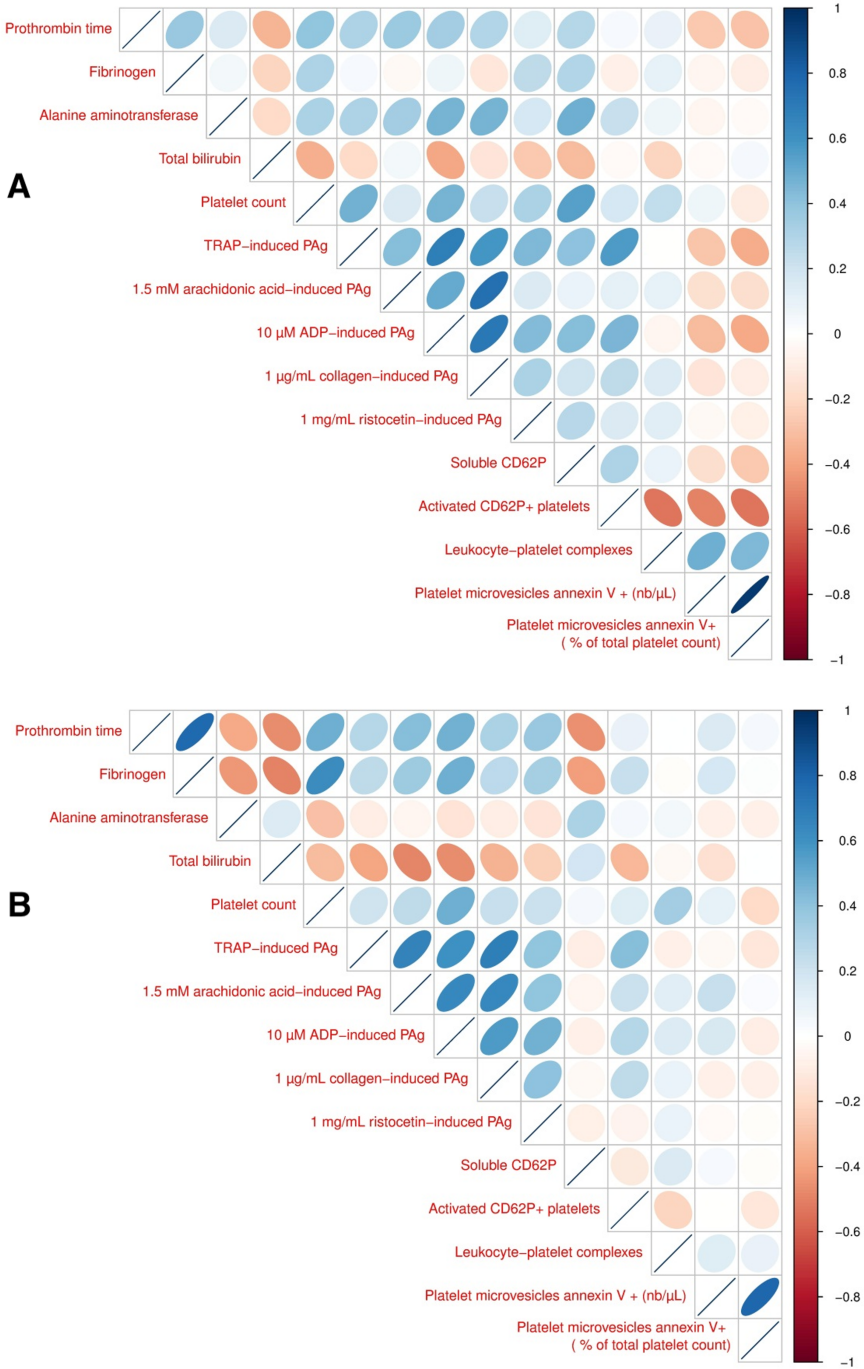
**Correlograms for repeated data (A) between subject correlation (static correlation) and (B) within subject correlation (dynamic correlation).** PAg: platelet aggregation. Comment: blue color indicates a positive correlation, red color indicates a negative correlation, color intensity and ellipse angulation indicate the strength of the correlation.

On the correlation between parameters of platelet functions (Fig 4A for static correlations and Fig 4B for dynamic correlations.

- The static correlation between platelet count and platelet aggregation was moderate but significant except for the two agonists collagen and arachidonic acid. About the dynamic correlation, a weak and positive association was found between platelet count and aggregation except for ADP that was more strongly linked, and same feature was found with parameters of platelet activation;
- The static correlation between parameters of platelet aggregation and activation found a positive and strong association between % of activated platelets CD62P positive and aggregations (ADP and TRAP) and with soluble CD62P. About dynamic correlation, the only valuable association was found between TRAP-induced aggregation and % activated platelets (positive and moderate-strong).

About the correlations between hepatic function and platelet function (Fig 4A and 4B)

- PT was significantly positively but moderately associated in level and in kinetic with platelet count and platelet aggregation in response to various agonists. Fibrinogen, considering the static correlation, was strongly, positively and significantly associated with platelet count. Concerning the dynamic correlation, a positive and strong association was found with platelet count, a positive and weak association was found with parameters of platelet aggregation except for ADP (more strongly).

### Clinical factor association

No association was found between the evolution of platelet count over time and clinical factor.

Considering the association with the parameters of platelet aggregation:

- Evolution of TRAP-induced platelet aggregation was associated with CHILD score (p-value = 0.04, CHILD C seems to have a slower increase), and UNOS score (p-value = 0.04, UNOS 4 seems to have a slower increase);
- Evolution of arachidonic acid-induced platelet aggregation was only associated with baseline creatinine (p-value = 0.04, high value of baseline creatinine was associated with a slower increase) and an history of thrombosis (p-value = 0.009, with higher values in preoperative and postoperative and a plateau effect at day 28);
- Evolution of collagen-induced platelet aggregation was associated with hemorrhagic history (p-value = 0.04, positive history seems to be associated with a decrease while the absence with an increase);
- Evolution of ristocetin-induced platelet agglutination was associated with CHILD score (p-value = 0.012, CHILD C seems to have a lower increase).

Noteworthy, the 3 patients having experienced a deep venous thrombosis had normal platelet count and aggregation profiles at D28.

## Discussion

The present study was designed to analyze evolution of platelet functions after liver transplantation in a prospective observational study. No prior sizing was done because of the lack of data available to feed the assumptions. Furthermore, we can assess an a posteriori sizing according observed value of ADP10 and a raise of 30% at day 28. Therefore, a sample size of 50 will have had 99% power to detect a difference in means of -16,100 (e.g. a first condition mean, of 53,600 and a second condition mean, of 69,700), assuming a standard deviation of differences of 22,500, using a paired t-test with a 0,050 two-sided significance level.

Within a 3-year period we analyzed results of 50 out of 101 patients scheduled for liver transplantation. Median age of patients was higher than in the literature (around 52 years) and only 8 women were included (8/50 compared to 43.4% in France according to French Biomedecine Agency, 2012). Nevertheless, clinical conditions of patients were similar to those published.

Because clinical thrombotic or hemorrhagic events after LT are seldom, it was not possible to evaluate the correlation of these events with platelet count and function; therefore, we only considered a previous history of thrombosis or bleeding. In humans, a low platelet count following partial hepatectomy or living donor liver transplantation has been shown strongly correlated with liver dysfunction and post-operative mortality [18,19]. Although the relation of causality between platelet count and clinical complication is very difficult to establish in humans, several experimental studies in animals demonstrated the role of platelets in liver regeneration after a partial liver resection [20,21]. However, most of these studies were performed in animals or humans with normal liver and evaluated platelet count more than platelet functions.

A huge number of data have been provided about platelet functions in liver disease, but only few exist on time course of platelet function recovery one month after transplantation. Indeed, whereas most papers concerning liver transplantation have studied platelet functions during and just after (maximal 10 days) surgery, the originality of the present work was to conduct a comprehensive analysis of platelet functions at 3 time-points up to 28 days post transplantation. As expected, there was a significant rise in platelet count, in line with CD34+ hematopoietic progenitor cell mobilization. Although the importance of stem cell mobilization is most likely to reflect the bone marrow regenerative pathway, as CD34 cell count correlated with platelet count, it might also have a beneficial effect. Interestingly, a recent study has treated patients with decompensated liver cirrhosis with autologous GM-CSF-mobilized peripheral blood CD34+ cells obtained after apheresis. Cells administered through the hepatic artery were well tolerated. After a few weeks, serum albumin was significantly increased and Doppler ultrasound showed a significant increase in blood flow velocity and volume [22], suggesting a beneficial role for CD34+ cells in slowing the decline of liver function in these patients. It would be interesting to analyze these cell populations in the long-term to correlate with liver function.

Although pre-LT platelet count was found associated with platelet aggregation tests, we describe a significant improvement of platelet aggregation along time post-LT, independently on platelet count. Indeed, we chose to adjust in most cases the number of platelets in the PRP used for aggregation assays to that of the first test (before the surgery). However, it would have been interesting to study platelet function 6 months after LT because, despite a global platelet aggregation recovery, two groups of subjects were still observed at 1 month: those having a normal platelet response and those with values remaining below reference range, representing about one-third of patients and not always those with the lowest platelet count. The influence of the toxic plasmatic environment itself on platelet functions in patients with liver cirrhosis should be evaluated in a further study by cross-experiments using washed platelets and normal or cirrhotic patient plasma. However, no difference in platelet function was found in multivariate analysis between patients transplanted for decompensated liver cirrhosis and patients transplanted for carcinoma, in whose liver function is much better, and therefore plasma toxic components lower.

Along with recovery of platelet function, no platelet activation was denoted. First, we reproduce previous data showing the absence of increase in circulating levels of platelet microvesicles in patients with cirrhosis [23,24], suggesting that PMV are not likely to be involved in the procoagulant imbalance associated with liver disease. Second, our results at D28 were close to those found in healthy subjects [23] using a similar method. Second, although platelet count dramatically increased by 3-3-fold, there was no increase in the percentage of leukocyte-platelet aggregates, most of patients remaining within the normal threshold of 20%. Finally, there was no evidence of platelet hyperactivity, as soluble CD62P tended to decrease at day 28. However, we cannot exclude an improvement of liver clearance of this marker. Even if soluble CD62P is a recognized marker of *in vivo* platelet activation [25], is not fully specific as it is also a marker of endothelial activation. Furthermore, we did not observe either an increase in soluble CD40L, a specific marker of platelet activation. Nevertheless, our results are in agreement with Pereboom et al, which showed no systemic platelet activation during or up to 10 days after liver transplantation in a similar number of patients, and by using tests complementary to ours [26]. In summary, platelets increased in number and function, without any biological signs of hyperactivation, within the limitation of chosen prothrombotic markers.

Our results are consistent when considering the associations between biological markers and clinical presentation. Indeed, an association was found between platelet function recovery (TRAP-and ristocetin-induced aggregation), but platelet count, and CHILD score. Thus, as Tripodi et al [27], previously suggested, patients with end-stage liver disease could be at bleeding risk, not solely because of portal hypertension but because different type of platelet dysfunction.

Even if the number of included patients was low, it allowed showing interesting biologic and clinical correlations. However, it was first an observational study on platelet function evolution in patients with liver cirrhosis before and after liver transplantation. It showed an increase in platelet function after LT, independently of the increase in platelet count, with the retrieve of the pathologic liver and the replacement with a safe one.

## Supporting information

S1 Table Comparison of aggregations between patients (N = 28) with a normal platelet count in plasma-rich platelets at D28 and 48 healthy controls. (DOCX)

## Abbreviations

HCC: hepatocellular carcinoma
ASAT: aspartate aminotransferase
ALAT: alanine aminotransferase
LC: Liver cirrhosis
LT: Liver transplantation
PMVs: platelet microvesicles
PRP: platelet-rich plasma
PPP: platelet-poor plasma
PLC: platelet-leukocyte complexes
PT: prothrombine time
