# Supplementary material for: Evolution of platelet functions in cirrhotic patients undergoing liver transplantation: A prospective exploration over a month
Source: PLoS One. 2018 Aug 2;13(8):e0200364. doi: 10.1371/journal.pone.0200364 (PMC6072007; doi:10.1371/journal.pone.0200364)
Supplement: S1 Table — (DOCX) [file pone.0200364.s001.docx]

**Supplemental Table.** Comparison of aggregations between patients (N=28) with a normal platelet count in plasma-rich platelets at D28 and 48 healthy controls.

| **Agonist** | **Maximal aggregation %**  median[Q1 ; Q3] | | **Wilcox. Test**  **P value** | **Adjusted pvalue - Bonferroni** |
| --- | --- | --- | --- | --- |
|  | **Controls** | **Patients at D28** |  |  |
| 5µM ADP | 84.5 [77;90.25] | 69.5 [61.5;79] | <0.0001 | 4,00E-04 |
| 10µM ADP | 84.5 [80.5;94] | 77 [64;81] | 2,00E-04 | 0.0028 |
| 20µM TRAP | 84 [81;90.5] | 68.5 [55.25;74.5] | <0.0001 | <0.0001 |
| 1.5 mM Arachidonic acid | 85.5 [82;89.75] | 72 [45;76.25] | <0.0001 | <0.0001 |
| 1 µg/mL Collagen | 89.5 [82;95] | 68 [35.25;75.25] | <0.0001 | <0.0001 |
| 1 mg/mL Ristocetin | 88.5 [82.75;96] | 81.5 [73.5;88.25] | 0.0023 | 0.0271 |

Data are expressed as median values [1^st^-3^rd^ interquartiles].

Controls are 48 healthy subjects of mean age 33±13 years.

Analysis compared data from controls and a subgroup of 28 among the 50 patients at D28, including only those having a platelet count in PRP above 150 G/L.
